# A Case of Chronic Undiagnosed 2:1 Isolated Atrial Flutter in a Patient With an Autoimmune Disease

**DOI:** 10.7759/cureus.48240

**Published:** 2023-11-03

**Authors:** Tegan Clarke, Eric Leung, Anthony Fleg

**Affiliations:** 1 Surgery, University of New Mexico School of Medicine, Albuquerque, USA; 2 Internal Medicine, University of New Mexico School of Medicine, Albuquerque, USA; 3 Family and Community Medicine, University of New Mexico School of Medicine, Albuquerque, USA

**Keywords:** chronic autoimmune disease, primary medical care, cardiovascular screening, cardiac arrythmia, atrial flutter with rapid ventricular response

## Abstract

Atrial flutter (AFL) is typically associated with structural heart diseases or metabolic abnormalities. However, isolated symptomatic AFL, which occurs without abnormal heart anatomy, remains a rare occurrence and is underrepresented in the literature. This case report highlights the significance of recognizing and investigating symptoms suggestive of arrhythmias, especially in patients with autoimmune conditions.

## Introduction

 Atrial flutter (AFL) affects approximately 200,000 new patients each year, making it the second most common cardiac arrhythmia after atrial fibrillation. However, the literature specifically focusing on AFL in the absence of structural cardiac abnormalities or in patients with comorbid autoimmune disease remains limited. Typically, AFL is observed in patients with underlying structural heart diseases or metabolic abnormalities [1-3]. These patients often have increased atrial size or other structural abnormalities before the symptomatic presentation of AFL [4]. Cases of symptomatic AFL without abnormal heart anatomy, termed isolated AFL, as presented in this case, are rare and poorly represented in the literature. One epidemiological study found isolated AFL in only three of 58,820 patients [1]. We present a case of unrecognized AFL in a patient with a history of an autoimmune disease to address the paucity of isolated atrial fibrillation literature.

## Case presentation

The patient is a woman in her mid-60s with a medical history of systemic lupus erythematosus (SLE) and antiphospholipid syndrome. She has no history of smoking, heavy alcohol use, or other substance use. She stays active by swimming and walking. Her body mass index (BMI) is 27.

During a regularly scheduled primary care visit, the patient reported an episode from one week prior, in which she suddenly felt flushed, nauseous, and lightheaded, accompanied by an episode of emesis. These symptoms persisted for two hours. Notably, she denied chest pain, shortness of breath, or anxiety during the episode. In addition, the patient mentioned occasionally experiencing similar episodes over the years. Her heart rate at this visit was 96, higher than the low 80s during prior visits.

At a subsequent primary care visit three months later, the patient described more than a month of persistently elevated heart rate. She had measured her heart rate using a home monitor several times in the previous few weeks and noted it in the range of 130s to 140s. At this visit, her seated heart rate was measured at 129 beats per minute. Further inquiry revealed the patient experienced similar episodes intermittently for more than 40 years. Approximately two decades ago, she was prescribed metoprolol for a “fast heart rate,” although she was never informed of having an arrhythmia nor received further workup. She had been symptom free and had not consulted a cardiologist in the last 20 years.

**Figure 1:**
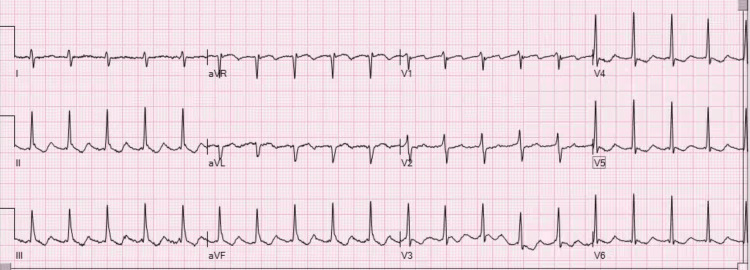
ECG taken at the second primary care visit. Positive flutter waves are noted in the inferior leads with negative flutter waves in V1. ECG: electrocardiogram

During the patient’s second visit, an electrocardiogram (ECG) showed clockwise typical AFL with 2:1 atrioventricular (AV) conduction (Figure 1). Consequently, anticoagulation was initiated and a cardiology referral was placed. A transthoracic echocardiogram completed two days later revealed no apparent structural or functional abnormalities. Thyroid-stimulating hormone (TSH) levels were within normal limits. An additional ECG completed two weeks later confirmed AFL.

**Figure 2:**
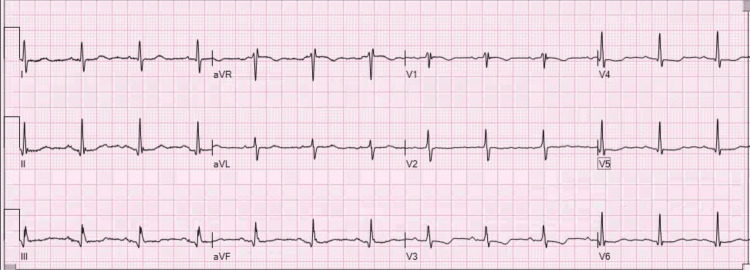
ECG taken at the follow-up cardiology visit after successful cardioversion with the restoration of the sinus rhythm ECG: electrocardiogram

Following four weeks of anticoagulation therapy, the patient underwent direct current (DC) cardioversion with a successful restoration of normal sinus rhythm (Figure 2). She continues to be followed by both her primary care team and cardiology.

## Discussion

AFL can manifest with a wide spectrum of symptoms, ranging from asymptomatic to symptoms related to the underlying arrhythmia, such as palpitations, lightheadedness, fatigue, exercise intolerance, and shortness of breath. Symptoms are typically interment and associated with rapid right ventricular contraction. 

Classically, AFL is a reentrant tachycardia initiated by an ectopic atrial beat [2]. Although the exact mechanism is unclear, data suggest that AFL involves a macro-reentrant tachycardia with a reentrant wave utilizing two electrical boundaries and congruous cycle length, leading to slower conduction circuits within the right atrium and myocardial remodeling [5,6]. Consequently, AFL is commonly observed in patients with underlying structural diseases, such as chronic obstructive pulmonary disease, pulmonary hypertension, and heart failure. Increased atrial size or other structural abnormalities have typically developed before symptomatic presentation when underlying AFL is present [4]. Notable risk factors include male sex, advancing age, hypertension, diabetes, history of alcohol abuse, and elevated BMI [1-3].

In addition, AFL is associated with underlying autoimmune diseases. Specifically, patients with SLE are nearly twice as likely to develop atrial fibrillation or AFL (confidence interval (CI) 1.43-2.24) [7]. Although the underlying pathogenesis is not fully understood, emerging evidence suggests the role of chronic inflammation. This relationship, applicable to this patient presentation, underscores the importance of cardiac evaluation in patients with autoimmune diseases.

The first-line management of AFL includes rate control utilizing calcium channel blockers or beta-blockers, which are highly effective in converting AFL to sinus rhythm. However, the treatment efficacy requires confirmation with follow-up ECG. In addition, isolated AFL carries an increased risk for subsequent development of atrial fibrillation and is associated with a similar stroke risk [4]. Therefore, anticoagulation management remains a Class 1A recommended cornerstone of management [8]. Considering the risk of thrombus formation due to the arrhythmia itself and potential for thromboembolic complications when initiating rate control with chemical cardioversion, cardiology consultation is essential for evaluation and treatment initiation.

## Conclusions

This case provides several noteworthy insights. First, this patient had experienced episodic tachycardia for more than 40 years and was prescribed metoprolol 20 years before presentation but was never evaluated for potential underlying etiology. Second, transthoracic echocardiogram showed no structural or functional abnormalities, which is atypical in patients with symptomatic AFL. Lastly, this patient had a history of autoimmune conditions, which have been well associated with increased rates of better-studied atrial fibrillation, but the literature on AFL is less clear.

In conclusion, this case report emphasizes the importance of a thorough evaluation and investigation of symptoms suggestive of arrhythmias, even if they occur intermittently or are mild, to ensure the long-term cardiac health of patients. Moreover, this underscores the challenges of chronic rate control with only pharmacologic agents without regular monitoring. Lastly, it highlights the importance of regular cardiovascular screening and follow-up care, especially in patients with autoimmune conditions.
